# Variation in Thermal Tolerance and Its Relationship to Mitochondrial Function Across Populations of *Tigriopus californicus*

**DOI:** 10.3389/fphys.2019.00213

**Published:** 2019-03-15

**Authors:** Alice E. Harada, Timothy M. Healy, Ronald S. Burton

**Affiliations:** Marine Biology Research Division, Scripps Institution of Oceanography, University of California, San Diego, La Jolla, CA, United States

**Keywords:** local adaptation, ATP synthesis, intertidal, timescale, ectotherm, copepod, latitudinal gradient

## Abstract

Variation in thermal tolerance plays a key role in determining the biogeographic distribution of organisms. Consequently, identifying the mechanistic basis for thermal tolerance is necessary for understanding not only current species range limits but also the capacity for range limits to shift in response to climate change. Although variation in mitochondrial function likely contributes to variation in thermal tolerance, the extent to which mitochondrial function underlies local thermal adaptation is not fully understood. In the current study, we examine variation in thermal tolerance and mitochondrial function among three populations of the intertidal copepod *Tigriopus californicus* found across a latitudinal thermal gradient along the coast of California, USA. We tested (1) acute thermal tolerance using survivorship and knockdown assays, (2) chronic thermal tolerance using survivorship of nauplii and developmental rate, and (3) mitochondrial performance at a range of temperatures using ATP synthesis fueled by complexes I, II, and I&II, as well as respiration of permeabilized fibers. We find evidence for latitudinal thermal adaptation: the southernmost San Diego population outperforms the northernmost Santa Cruz in measures of survivorship, knockdown temperature, and ATP synthesis rates during acute thermal exposures. However, under a chronic thermal regime, survivorship and developmental rate are more similar in the southernmost and northernmost population than in the mid-range population (Abalone Cove). Though this pattern is unexpected, it aligns well with population-specific rates of ATP synthesis at these chronic temperatures. Combined with the tight correlation of ATP synthesis decline and knockdown temperature, these data suggest a role for mitochondria in setting thermal range limits and indicate that divergence in mitochondrial function is likely a component of adaptation across latitudinal thermal gradients.

## Introduction

Environmental temperature is one of the most influential abiotic factors in shaping the performance and survival of organisms (Hochachka and Somero, 2002). Upper and lower thermal limits of populations and species typically increase from the poles to the equator (Sunday et al., 2011), and in general, maximum habitat temperatures at equatorward range limits closely match maximum tolerated temperatures (Sunday et al., 2012). Temperatures that limit organismal performance also often align with habitat temperatures that occur over prolonged periods (i.e., seasons) (Johnston and Dunn, 1987; Pörtner, 2010). Together, these observations suggest that variation in temperature and upper thermal tolerance play key roles in determining the biogeographic distribution of organisms (Pörtner, 2010; Sunday et al., 2011, 2012; Deutsch et al., 2015). Thus, identifying the mechanistic basis for differences in upper thermal limits among species and populations is necessary to understand not only current species range limits but also the capacity for range limits to shift as a result of local genetic adaptation and phenotypic plasticity in response to natural or anthropogenic climate change (Pörtner et al., 2006; Pritchard and Di Rienzo, 2010; Sunday et al., 2012; Seebacher et al., 2015; Bay et al., 2017; Healy et al., 2018).

Mitochondria play a role in a diversity of cellular functions (e.g., calcium signaling, cell growth and differentiation, cell cycle control, and cell death), and their central role in energy metabolism suggests that mitochondria may be intimately associated with thermal performance and tolerance in ectotherms. For instance, the loss of mitochondrial ATP synthesis capacity in fish hearts is thought to occur at temperatures immediately below tolerance limits during acute exposures to high temperatures (Iftikar and Hickey, 2013; Christen et al., 2018; O’Brien et al., 2018). In contrast, in some species, the loss of whole organism tolerance occurs at temperatures below those resulting in decreased mitochondrial oxidative capacity (e.g., see Dahlhoff et al., 1991; Dahlhoff and Somero, 1993). In any case, relative changes in mitochondrial oxidative phosphorylation and proton leak at sublethal temperatures may underlie thermal limits for whole-organism aerobic capacity and variation in aerobic capacity across temperatures (e.g., Pörtner, 2001). In addition, mitochondrial functions are known to respond to temperature both as a result of phenotypic plasticity and local genetic adaptation through changes in mitochondrial amount (Egginton and Johnston, 1984; Orczewska et al., 2010; Dhillon and Schulte, 2011; O’Brien, 2011), oxidative capacity (Guderley, 2004; Kraffe et al., 2007; Grim et al., 2010; Seebacher et al., 2010; Chung and Schulte, 2015; Chung et al., 2017a), oxygen affinity (Chung et al., 2017b), membrane composition (Hazel, 1995; Kraffe et al., 2007; Grim et al., 2010; Chung et al., 2018a), and enzyme activities and amounts (St-Pierre et al., 1998; Guderley, 2004; McClelland et al., 2006; LeMoine et al., 2008; Orczewska et al., 2010), which clearly suggest that modulation of mitochondrial functions is an important component of cellular responses to temperature change. Thus, although variation in mitochondrial functions likely contributes to the mechanistic basis for variation in thermal tolerance, neither the role of mitochondrial mechanisms across different timescales of thermal exposure (i.e., acute versus chronic) nor the extent to which variation in mitochondrial functions underlies local thermal adaptation across environmental temperature gradients is fully understood.

**Figure 1:**
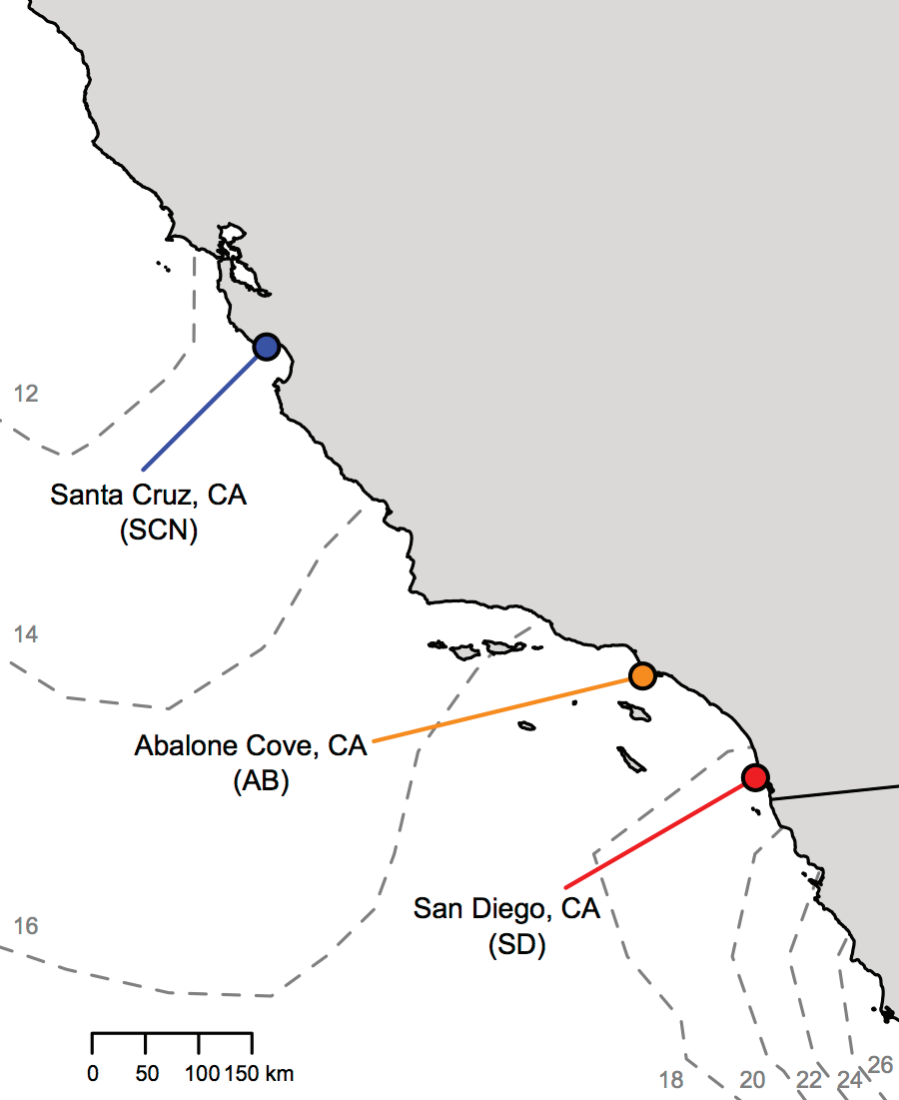
Locations of *T. californicus* collection sites along the coast of California (gray = land; white = Pacific Ocean). Sites are indicated by colored circles [red = San Diego (SD); orange = Abalone Cove (AB); blue = Santa Cruz (SCN)]. Dashed gray lines outline contours of average sea surface temperatures from the first week of July 2017 with temperature in degrees Celsius indicated by small gray numbers (obtained from: http://www.cpc.ncep.noaa.gov/products/GIS/GIS_DATA/sst_oiv2/index.php).

In the current study, we examine variation in thermal tolerance and mitochondrial function among three populations of the intertidal copepod *Tigriopus californicus* found across a latitudinal thermal gradient along the coast of California, USA (Figure 1). *T. californicus* inhabits high-intertidal splash pools on the west coast of North America from Baja California, Mexico, to Alaska, USA, and is an ideal candidate species to study local genetic adaptation of both thermal tolerance and mitochondrial functions. Gene flow among populations of *T. californicus* is remarkably low even over short distances (Burton, 1998; Willett and Ladner, 2009; Peterson et al., 2013), and consequently, there are high levels of genetic divergence among populations both in genes encoded in the mitochondrial genome and in genes in the nuclear genome that encode products that function in the mitochondria (e.g., 9.5–26.5% sequence divergence in the mitochondrial genome among populations with Fst ≈ 0.98 across a large number of populations; Edmands, 2001; Peterson et al., 2013; Pereira et al., 2016; Barreto et al., 2018). Furthermore, there is substantial evidence for local adaptation of upper thermal tolerance among populations of *T. californicus,* with more southern populations generally able to tolerate higher temperatures than more northern populations (Willett, 2010; Kelly et al., 2012; Tangwancharoen and Burton, 2014; Hong and Shurin, 2015; Pereira et al., 2017; Leong et al., 2018; Willett and Son, 2018).

Differences in mitochondrial genotype and function have been linked to variation in thermal performance in species of *Drosophila* (Pichaud et al., 2012; Hoekstra et al., 2013); however, previously published studies in *T. californicus* have not directly addressed this potential mechanistic relationship. It has been observed that disruption of mitochondrial functions in inter-population F_2_ hybrids of *T. californicus* does not result in decreased thermal tolerance (Willett, 2012; Pereira et al., 2014), despite lower mitochondrial ATP synthesis capacities at 20°C (Ellison and Burton, 2008). However, ATP synthesis capacity has not been measured during exposures to high temperatures in *T. californicus,* and thus it is possible that mitochondrial dysfunction may play a role in determining variation in upper thermal tolerance in this species in general. To investigate the mechanistic relationships between upper thermal tolerance and mitochondrial function in *T. californicus* and how these relationships may contribute to latitudinal thermal adaptation across timescales (i.e., acute versus chronic or within-generation versus across-generations), here we assess (1) intraspecific variation in upper thermal tolerance and performance among populations using survivorship of heat stresses from 34 to 37°C, knockdown temperatures, developmental survivorship, and developmental rate at 20 and 25°C, (2) inter-population variation in mitochondrial ATP synthesis capacity and thermal sensitivity of ATP synthesis rates during acute thermal exposure, and (3) variation in mitochondrial oxidative and leak respiration rates among populations at both intermediate and high temperatures.

## Materials and Methods

### Copepod Collection and Culture

*Tigriopus californicus* were collected from high rocky tide pools at three locations in California, USA (Figure 1): “SD” from Ocean Beach, San Diego County (32° 45′ N, 117° 15′ W); “AB” from Abalone Cove, Los Angeles County (33° 44′ N, 118° 22′ W); and “SCN” from Santa Cruz County (36° 56′ N, 122° 02′ W). These populations are found across a latitudinal gradient in temperature along ~775 km of the coast of California with about a 6°C difference in sea surface temperature (Figure 1) and ~4°C difference in mean annual air temperatures (e.g., Pereira et al., 2017) from SD to SCN.

Copepods were kept in multiple 400-ml beakers containing 250-ml filtered seawater (0.4 μm, 35 ppt). Cultures were fed ad libitum with a combination of ground TetraVeggie algae wafers and powdered Spirulina. Prior to use in any thermal exposure assays, cultures were maintained in the laboratory for at least 1 month (one generation) at 20°C with a 12-h light/dark cycle.

### Survivorship of Acute Heat Stress Across Temperatures

Tolerance of a 1-hour heat stress at different temperatures was measured using methods similar to those previously described for *T. californicus* (Willett, 2010; Kelly et al., 2012; Tangwancharoen and Burton, 2014; Pereira et al., 2017; Leong et al., 2018; Willett and Son, 2018). In brief, groups of 10 adult copepods from each population were removed from stock cultures by glass pipette and transferred to separate 15-ml Falcon™ tubes (Thermo Fisher Scientific, Waltham, MA) containing 10-ml of 20°C filtered seawater. After 10 min, the tubes were submerged in a preheated water bath (Julabo USA Inc., Allentown, MA) at one of four temperatures: 34, 35, 36, or 37°C. Following 1 h of heat stress, tubes were moved to a beaker containing 20°C water for an additional hour to allow the temperature inside the tubes to gradually decrease back to holding conditions. Copepods were then transferred to a fresh 10-cm petri dish containing 20°C filtered seawater. Tolerance of the acute high temperature exposures was assessed after 3 days as the proportion of surviving individuals in each group of 10 (*n* = 6 per population and temperature).

### Knockdown Temperature

Maximum tolerated temperature during a ramping heat stress was assessed by knockdown temperature (i.e., the temperature at which movement and responsiveness cease; e.g., Gilchrist and Huey, 1999; Hoffmann et al., 2003). For each trial, eight adult copepods from each population were individually pipetted from stock cultures into 0.2-ml strip tubes (without caps) and carryover water was replaced with 100 μl of 20°C filtered seawater. After approximately a 10-min recovery, the strip tubes were placed in an Applied Biosystems SimpliAmp™ Thermal Cycler (Thermo Fisher Scientific, Waltham, MA). Locations of individuals from each population were randomized in the thermal cycler for each trial. The heat stress regime utilized the AutoDelta function of the thermal cycler with the following protocol: 20°C for 5 min, +0.1°C every 20 s from 20 to 32°C, and +0.1°C every 60 s from 32 to 45°C. The thermal cycler lid was left open during the temperature ramp and copepods were monitored continuously from above. Once a copepod stopped responding to gentle tapping of their tube, loss of responsiveness was assessed by cycling 40 μl of the water in the tube with a micropipette taking care not to touch the copepod directly. In general, this manipulation results in the observation of active swimming behavior in *T. californicus*; however, at high temperatures, this response is no longer observed and copepods gradually sink to the bottom of the tubes passively. If a copepod actively responded to the water movement, responsiveness tests were paused and monitoring continued. If no active response was observed, the responsiveness test was repeated up to three times (total time for end-point determination for an individual was ~6 s). The first temperature at which a copepod did not respond to three successive tests was recorded as the individual’s knockdown temperature (*n* = 16 per population over two trials). After this end point was determined, individuals were transferred by glass pipette to recovery 10-cm petri dishes containing 20°C filtered seawater (one per population); survivorship 1 day after the assay was >90%.

### Developmental Survival and Rate

Gravid females with mature (red) egg sacs were removed from stock cultures by glass pipette and immobilized on filter paper (*n* = 24 per population). Egg sacs were dissected from the females with a needle and placed in 6-well plates containing 20°C filtered seawater (one egg sac per well). Eggs were allowed to hatch overnight at 20°C, and in the morning, the nauplii (i.e., offspring) from each sac were counted and split between two 6-well plates (one well per plate per egg sac). The two plates were incubated for 21 days at 20 or 25°C (one plate per temperature). Spirulina was added to each well twice per week as a food source, and salinity was adjusted using addition of deionized water to compensate for evaporation weekly. On days 7, 14, and 21, surviving individuals and the number of nauplii that had metamorphosed into copepodids were counted in each well. The number of wells in which adult males were observed was also recorded. The presence of adult males, rather than females, was used, because the final molt to adult male *T. californicus* results in visually conspicuous antennae that are used in mating behaviors (Burton, 1985; Tsuboko-Ishii and Burton, 2017). In contrast, young adult females are visually difficult to distinguish from juvenile females and males until later stages of maturity (Burton, 1985) and thus are suboptimal indicators of developmental stage.

For measurement of developmental rate at 20 and 25°C, mature egg sacs were dissected and split post-hatch as described above (*n* = 18, 16, and 18 for SD, AB, and SCN, respectively), but in this case split egg sacs were pooled into 10-cm petri dishes (one dish per population and temperature) and developmental stage was monitored daily. The rate of development was tracked by the day of appearance of copepodids in the dish and was scored for each individual (Tangwancharoen and Burton, 2014). Regardless of developmental temperature, the survivorship of offspring for all populations was 78.6–94.0%, and all surviving individuals metamorphosed by 14 days post clutch split.

### ATP Synthesis Rate

ATP synthesis assays were conducted using similar methods to those of Ellison and Burton (2006). Mitochondria were isolated from groups of 30 adult copepods per assay (*n* = 6 per population). Preliminary tests indicated that this number of copepods was sufficient to show linear ATP production over time for at least 1 h across the range of temperatures tested in this study (20 to 40°C). Copepods were rinsed with 200 μl ice cold homogenization buffer (400 mM sucrose, 100 mM KCl, 6 mM EGTA, 3 mM EDTA, 70 mM HEPES, 1% w/v BSA, pH 7.6) (Moyes et al., 1985) and homogenized on ice in 800 μl homogenization buffer with a teflon on glass homogenizer. The homogenate was transferred to 1.5-ml microcentrifuge tubes (Eppendorf, Hamburg, Germany) and centrifuged at 1,000 g for 5 min at 4°C. The supernatant was transferred to new microcentrifuge tubes and was centrifuged at 11,000 g for 10 min at 4°C. The supernatant resulting from this second centrifugation was then removed and the pellet was resuspended in 255 μl assay buffer (560 mM sucrose, 100 mM KCl, 10 mM KH_2_PO_4_, 70 mM HEPES, pH 7.6; modified from Moyes et al., 1985).

Mitochondrial isolations were split into ten 25-μl aliquots in PCR tubes for ATP synthesis assays at nine incubation temperatures (one tube per temperature: 20, 25, 30, 35, 36, 37, 38, 39, and 40°C, and one tube for measurement of initial ATP concentrations in the assays). The remaining 5 μl of isolate was used for protein content determination. To initiate synthesis assays, 5 μl of a substrate cocktail was added to each tube, and then the tubes were incubated at the desired temperature for 10 min in an Applied Biosystems SimpliAmp^™^ Thermal Cycler (Thermo Fisher Scientific, Waltham, MA). The substrate cocktail depended on the electron transport system (ETS) complexes that were used to drive electron transport and subsequent ATP synthesis. In our study, we measured ATP synthesis rate as a result of electron donation to complex I (CI; final substrate concentrations in assay: 5 mM pyruvate, 2 mM malate, and 1 mM ADP), complex II (CII; final substrate concentrations in assay: 10 mM succinate, 0.5 μM rotenone, and 1 mM ADP), and complex I and complex II in combination (CI&II; final substrate concentrations in assay: 5 mM pyruvate, 2 mM malate, 10 mM succinate, and 1 mM ADP). After the incubation period, 25 μl of each assay was added to an equal volume of CellTiter-Glo (Promega, Madison, WI), which both halts ATP synthesis and allows quantification of ATP concentration. Tubes used to measure initial ATP concentrations in the assays also had 5 μl of substrate cocktail, but CellTiter-Glo was added immediately following substrate addition. Therefore, these tubes accounted for any signal that was not a result of ATP synthesis during the assays. After a 10-min incubation at room temperature, the samples and controls were mixed by shaking, read on a luminometer, and compared with a set of ATP standards (5 nM to 10 μM prepared in assay buffer; 25 μl of each standard was mixed with 25 μl of CellTiter-Glo as described above for samples and controls). ATP synthesis rates were calculated after control values were subtracted from sample values, and rates were then normalized for protein content in the corresponding mitochondrial isolation using NanoOrange Protein Quantitation Kit assays (Thermo Fisher Scientific, Waltham, MA) according to the manufacturer’s instructions. Note that, while our buffer contained compounds (HEPES, sucrose, and potassium chloride) that have the potential to interfere with this assay at high concentrations (10, 10, and 20 mM, respectively), the final concentrations present after dilution were below these maxima for HEPES and potassium chloride (1.37 and 1.96 mM, respectively), and sucrose concentration was approximately the maximum recommended according to the manufacturer’s instructions (10.98 mM).

### High-Resolution Respirometry of Cell-Permeabilized Copepods

Respirometry was performed using a Clark-type electrode system (Oxygraph Plus System, Hansatech Instruments Ltd., England). The electrode was calibrated at either 20 or 35°C using air-saturated assay buffer (see “ATP synthesis rate” section above). Groups of 40 adult copepods were crudely homogenized using blue polypropylene pestles (Thomas Scientific, Swedesboro, NJ) in a 1.5-ml microcentrifuge tube to which 500 μl cold (~0°C) BIOPS was added (2.77 mM CaK_2_EGTA, 7.23 mM K_2_EGTA, 5.77 mM ATP, 6.56 mM MgCl_2,_ 20 mM taurine, 15 mM Phosphocreatine, 20 mM imidazole, 0.5 mM dithiothreitol, 50 mM K-MES, pH 7.1; Lemieux et al., 2011). Homogenized animals were then permeabilized by incubation in BIOPS containing 81.25 μg ml^−1^ saponin (concentration determined from Pichaud et al., 2011, and preliminary trials) for 30 min at 4°C prior to use in respirometry assays. After incubation, BIOPS was removed and 500 μl of assay buffer was added. The permeabilized copepods in assay buffer were then transferred into the respirometry chamber. The ETS was activated with CI substrates (5 mM pyruvate and 5 mM malate) and 2.5 mM ADP, after which state 3 respiration rate was measured (i.e., the maximal respiration rate under phosphorylating conditions; Scheffler, 2008). A 10 μM cytochrome c was then added to assess membrane integrity and in general resulted in no change in respiration rate. Next, 2 μg ml^−1^ oligomycin was added to measure state 4_ol_ respiration rate. State 4 is the rate of respiration in the absence of phosphorylation of ADP, which is the minimum rate required to counter proton leak and maintain proton motive force across the inner mitochondrial membrane; state 4_ol_ achieves this artificially with the addition of oligomycin, which inhibits complex V (Scheffler, 2008). Concentrations of substrates and inhibitors were modified from Pesta and Gnaiger (2012) based on preliminary trials.

### Statistical Analyses

All statistical analyses were performed in R v3.4.0 (R Core Team, 2017) using generalized linear models (GLM) and ANOVA followed by post-hoc tests with a threshold for statistical significance of α = 0.05. Differences in survival of 1-h acute heat stress were tested with population and exposure temperature as factors in a logistic GLM with a binomial error distribution. Pairwise comparisons among temperatures within populations and among populations within temperatures were conducted by t tests (paired or unpaired as appropriate) with a Bonferroni correction to determine significance. Data for knockdown temperatures and clutch sizes were analyzed by ANOVA with population as a factor followed by Tukey post-hoc tests. The same procedure was utilized to analyze mitochondrial respiration data, but population was added as an additional explanatory factor. Variation in time to metamorphosis (i.e., developmental rate) was assessed among treatments (populations x temperature) by Kruskal-Wallis ANOVA followed by Nemenyi tests. Significance of all pairwise comparisons was determined after a Bonferroni correction of alpha. Differences in developmental survival were calculated for each egg sac (25–20°C; see Table S1 for group means, metamorphosis and adult proportions) and were analyzed by mixed-effect linear models with fixed effects of population and day and a random effect of egg sac. Post-hoc comparisons were then performed with Tukey tests. Mixed-effect models were also used to assess variation in ATP synthesis rates with population and temperature as fixed effects and mitochondrial isolation as a random effect. Post-hoc pairwise comparisons for ATP synthesis rate were conducted similarly to those for survival of 1-h heat stress but with the use of the Benjamini-Hochberg method to correct for multiple comparisons (Benjamini and Hochberg, 1995). In all cases, interactions between factors were included in the fitted linear models. ANOVA tables for all statistical tests obtained from R are available in the supplementary materials (Tables S2–S12).

## Results

### Thermal Tolerance and Performance

**Figure 2:**
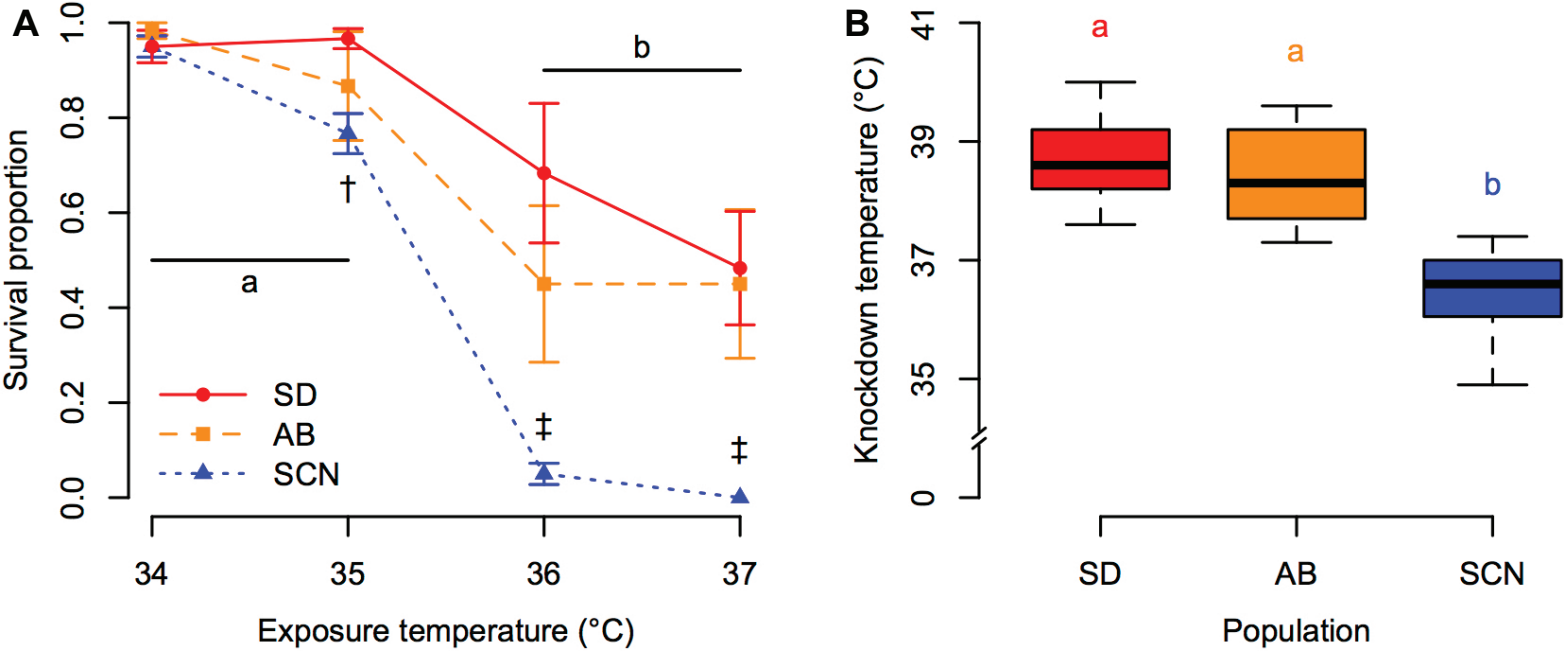
Acute upper thermal tolerance assessed by survivorship of 1-h heat stress **(A)** and knockdown temperature **(B)** in San Diego (SD; red, circles, solid lines), Abalone Cove (AB; orange, squares, dashed lines), and Santa Cruz (SCN; blue, triangles, dotted lines) copepods. **(A)** Data are presented as mean ± SEM; shared letters indicate temperatures that are not significantly different within populations; dagger symbols indicate differences among populations within a temperature († = SCN different from SD, ‡ = SCN different from SD and AB). **(B)** Data are presented as box plots; shared letters indicate populations that do not differ significantly.

Patterns of variation in tolerance of acute heat stress among populations of *T. californicus* were consistent regardless of the methods used to assess tolerance. Survivorship of 1-h heat stress was affected by a significant interaction between population and exposure temperature (*p* = 5.8 × 10^−5^; Figure 2A). Post-hoc comparisons detected significant declines in survivorship between 35 and 36°C in all populations (*p* ≤ 1.4 × 10^−5^). However, the survival proportion of SCN was lower than that of SD at 35°C (*p* = 6.9 × 10^−4^) and was lower than the survival proportions of both SD and AB at 36 and 37°C (*p* ≤ 9.1 × 10^−8^). This pattern of decreased upper thermal tolerance in the northern SCN population was also observed through variation in knockdown temperature among populations (*p* = 6.1 × 10^−11^; Figure 2B). For both measurements of acute upper thermal tolerance, there were slight trends for lower mean tolerance in AB copepods than in SD copepods, but these trends were not supported statistically after correction for multiple comparisons (1-h heat stress: *p* ≥ 9.5 × 10^−3^ compared to corrected α = 1.7 × 10^−3^; knockdown temperature: *p* = 0.63).

**Figure 3:**
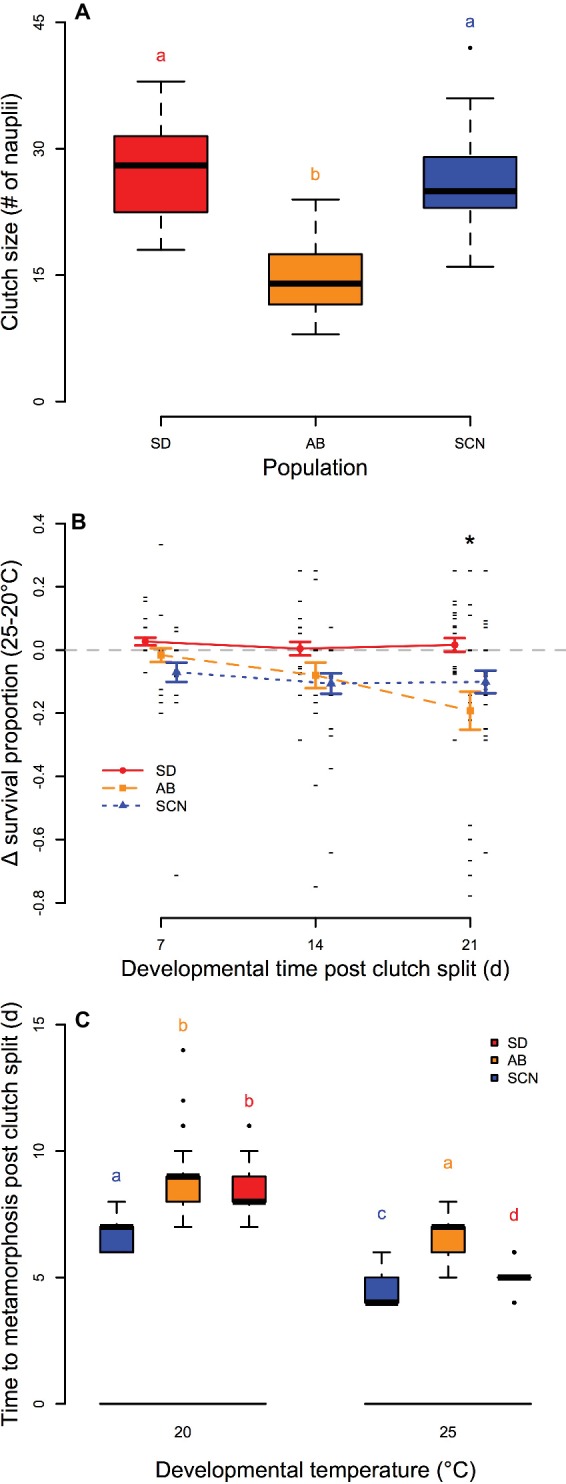
Egg clutch size **(A)**, survival of development at 25°C compared to 20°C **(B)**, and time to metamorphosis at 20 and 25°C **(C)** in San Diego (SD; red, circles, solid lines), Abalone Cove (AB; orange, squares, dashed lines), and Santa Cruz (SCN; blue, triangles, dotted lines) copepods. **(A,C)** Data are presented as box plots; shared letters indicate groups that are not significantly different. **(B)** Data are presented as mean ± SEM; small black dashes display difference for individual clutches; asterisk indicates a significantly lower difference in survival between 25 and 20°C at 21 days than at 7 or 14 days for AB and a significant difference between SD and AB at 21 days.

We observed significant variation in the number of eggs per clutch among populations (*p* = 1.0 × 10^−12^; Figure 3A) with smaller egg sacs from AB females than those from either SD or SCN females (*p* < 1.0 × 10^−4^). Differences in survival of offspring from split egg clutches over 3 weeks of development and early adulthood at 20 and 25°C were significantly affected by an interaction between population and time (*p* = 2.4 × 10^−3^; Figure 3B). In general, development at 25°C had either no effect or negative effects on survival compared to development at 20°C in all three populations. There was no variation in the relative effects of development at 25°C on survival with time in either SD or SCN (*p* ≥ 0.97), and there were trends for greater negative effects of 25°C on survival in SCN than in SD on all days, but these trends were not significant in post-hoc tests (*p* ≥ 0.23). In contrast, the difference in survival between 25 and 20°C in AB became more negative over the 3 weeks with a significant decrease in relative survival at 21 days compared to 7 or 14 days (*p* ≤ 1.7 × 10^−2^). Additionally, on day 21, the difference in survival between 25 and 20°C was significantly lower in AB than in SD (*p* < 1.0 × 10^−3^). Not surprisingly, there were also significant effects of temperature on developmental rate in all three populations with more rapid development at 25°C than at 20°C (*p* < 2.8 × 10^−11^ for all populations; Figure 3C), although the decrease in median time to metamorphosis varied among populations (7, 8, and 9 days post clutch split at 20°C to 4, 5, and 7 days post clutch split at 25°C for SCN, SD, and AB, respectively). There were also significant effects of population on time to metamorphosis at both temperatures: at 20°C, SCN developed faster than either SD or AB (*p* < 8.9 × 10^−11^ for both), and at 25°C, all three populations were significantly different from each other with fastest development in SCN, slowest development in AB, and intermediate developmental rate in SD (*p* ≤ 5.8 × 10^−3^). Overall, these results suggest modest negative effects of 25°C on development in *T. californicus*, which are generally more pronounced in AB than in the other two populations in our study. Furthermore, AB demonstrates lower performance (i.e., smaller clutch sizes or slower developmental rate) than SD or SCN even at 20°C.

### ATP Synthesis Rate and Mitochondrial Respiration

**Figure 4:**
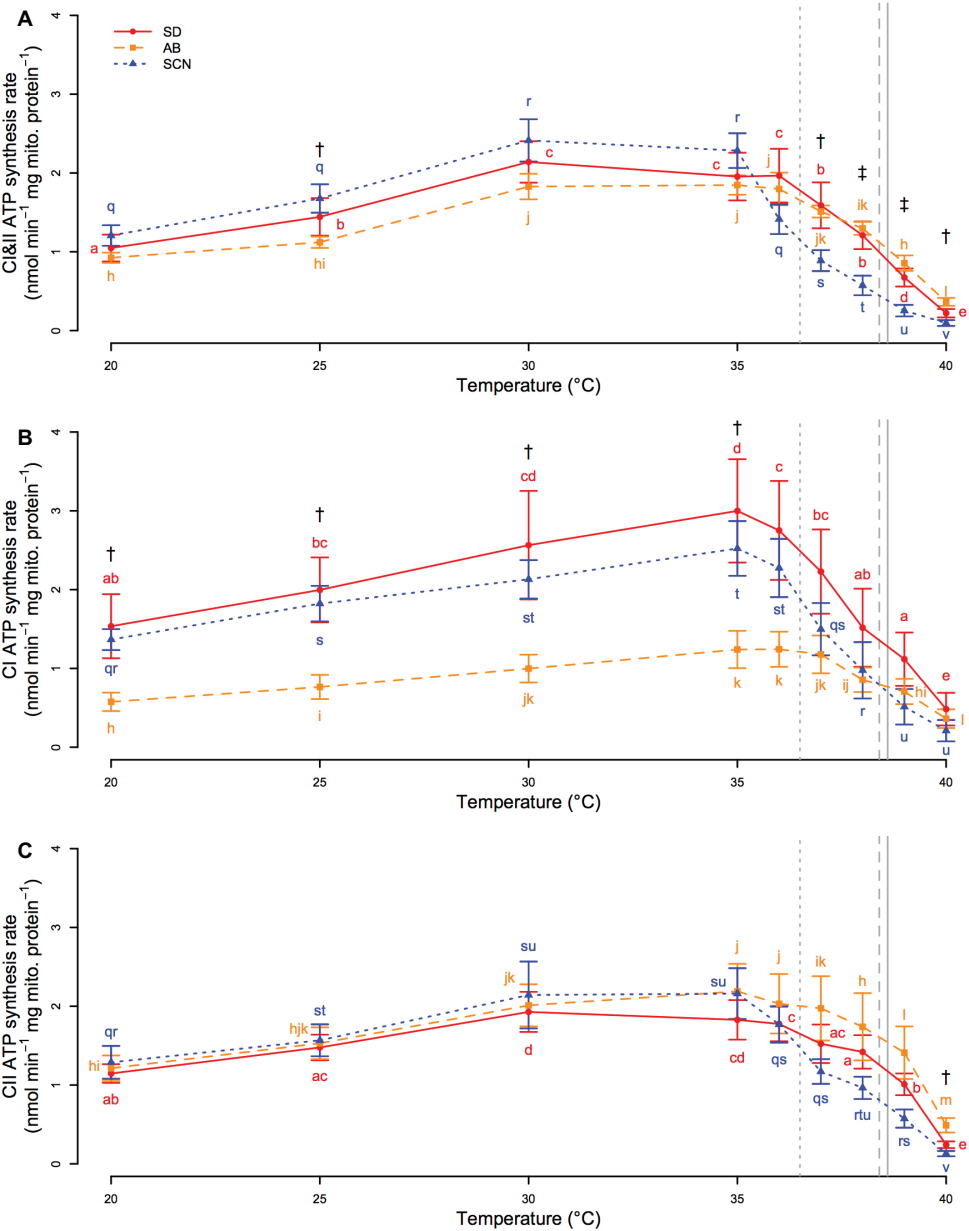
CI&II- **(A)**, CI- **(B)**, and CII-fueled **(C)** ATP synthesis rate from 20 to 40°C in San Diego (SD; red, circles, solid lines), Abalone Cove (AB; orange, squares, dashed lines), and Santa Cruz (SCN; blue, triangles, dotted lines) copepods. Data are presented as mean ± SEM. Letters indicate the results of post-hoc tests within populations. Daggers indicate temperatures at which there is a difference between SCN and AB. Double daggers indicate temperatures at which there is a difference between SCN and both AB and SD. Vertical gray lines display mean knockdown temperatures for each population (SD – solid; AB – dashed; SCN – dotted).

Regardless of the substrates and complexes used (CI, CII, or CI&II) to donate electrons to the ETS and drive ATP synthesis, synthesis rate was affected by significant interactions between population and temperature (*p* ≤ 5.1 × 10^−3^). If electrons were donated to both CI and CII (Figure 4A), ATP synthesis rate increased from 20 to 30°C in all three populations. Synthesis rate then plateaued from 30 to 35°C in SCN and from 30 to 36°C in SD and AB. Above these temperatures, ATP synthesis declined rapidly, and at temperatures greater than 36°C, rates in SCN were either lower than those in AB (*p* ≤ 8.2 × 10^−3^; 37 and 40°C) or lower than those in AB and SD (*p* ≤ 2.5 × 10^−2^; 38 and 39°C). SCN ATP synthesis rate was also significantly higher than AB ATP synthesis rate at 25°C (*p* = 3.8 × 10^−2^). If only CI substrates were used to drive ATP synthesis (Figure 4B), there were increases in synthesis rate with temperature from 20 to 35°C in all populations. At temperatures above 35°C, synthesis rate rapidly declined with temperature in SD and SCN, whereas in AB rates plateaued from 35 to ~37°C before declining at higher temperatures. As a result, there were no significant differences between the populations above 35°C (*p* ≥ 0.07). In contrast, CI-driven ATP synthesis was faster in SCN than in AB from 20 to 35°C (*p* ≤ 3.1 × 10^−2^), and similar trends were observed between SD and AB, although these trends were not detected as significant in post-hoc tests (0.051 < *p <* 0.108 for all). If only CII was used to drive electron transport (Figure 4C), patterns of change in ATP synthesis rate with temperature in all populations were similar to those described above for CI&II-fueled ATP synthesis. However, only one population effect on CII-driven synthesis rate was detected by post-hoc tests with a higher rate in AB than in SCN at 40°C (*p* = 2.5 × 10^−2^). Taken together, these results suggest that population- or temperature-mediated differences in CI&II ATP synthesis rate in *T. californicus* likely reflect contributions of population and temperature effects on synthesis rate when CI or CII are fueled separately, although under saturating conditions the independent CI and CII rates are not additive when the ETS is provided with both CI and CII substrates in combination. Additionally, our results indicate that AB synthesis rates, particularly when fueled through CI, are compromised relative to at least SCN rates from 20 to ~35°C, and that ATP synthesis capacity suffers high-temperature collapse at lower temperatures in SCN than in either SD or AB.

**Figure 5:**
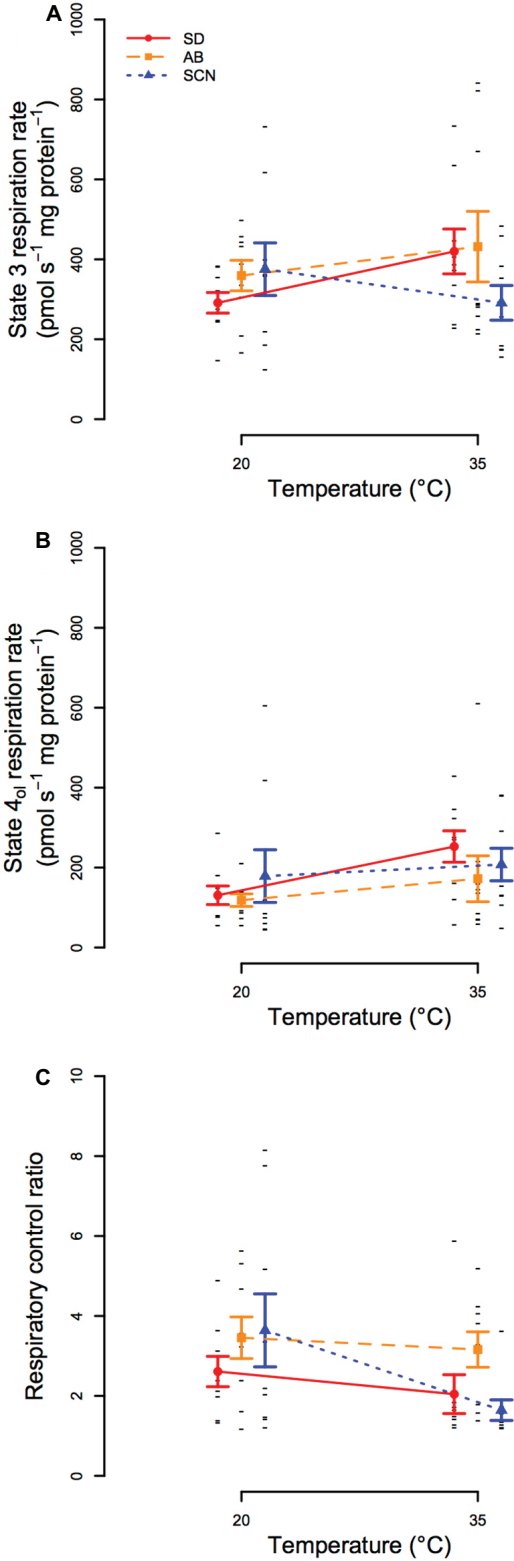
State 3 **(A)** and state 4_ol_ **(B)** mitochondrial respiration and respiratory control ratios (RCRs; **C**) at 20 and 35°C in San Diego (SD; red, circles, solid lines), Abalone Cove (AB; orange, squares, dashed lines), and Santa Cruz (SCN; blue, triangles, dotted lines) copepods. Data are presented as mean ± SEM, and small black dashes display values for each replicate. Significant effects of temperature for state 4_ol_ and RCR were detected by ANOVA, but post-hoc tests did not resolve any statistical differences among groups for all three traits.

In general, high-resolution respirometry experiments found that mitochondrial oxygen consumption rates fueled by CI substrates were variable within populations and temperatures in our study, and few differences were resolved statistically. State 3 respiration rate (Figure 5A) was not significantly affected by population (*p* = 0.47), temperature (*p* = 0.57), or an interaction between population and temperature (*p* = 0.21), and state 4_ol_ respiration rate (Figure 5B) and respiratory control ratio (RCR = state 3/state 4_ol_; Figure 5C) were also unaffected by population (*p* ≥ 0.19) or an interaction between population and temperature (*p* ≥ 0.25). There were significant main effects of temperature on state IV_ol_ oxygen consumption rate and RCR (*p* ≤ 3.6 × 10^−2^), but these effects were not resolved by post-hoc tests (within population *p* ≥ 0.11). The main effects of temperature that were detected may be a consequence of slight trends for increases in state 4_ol_ respiration rate and decreases in RCR from 20 to 35°C in all populations. Of these trends, the most notable is the decline in mean RCR in SCN at 35°C (post-hoc *p* = 0.11), which may be consistent with the rapid declines in ATP synthesis rate at temperatures above 35°C in SCN described above.

## Discussion

The results of the current study demonstrate intraspecific variation in both thermal tolerance and ATP synthesis capacity among allopatric *Tigriopus californicus* populations that are found across a latitudinal thermal gradient. Differences in tolerance of acute high temperatures among populations are generally consistent with variation in habitat temperature, whereas differences in chronic temperature effects among populations are less clearly correlated with environmental temperatures. Populations have temperature-dependent differences in ATP synthesis capacity, as revealed by variation in ATP synthesis rates in isolated mitochondria across temperatures. Furthermore, inter-population differences in loss of synthesis capacity during acute exposure to high temperatures parallel the differences in acute upper thermal tolerance, and variation in ATP synthesis rate among populations at 25°C is potentially consistent with differences in effects of chronic exposure to 25°C on development as well. These results suggest that there are evolved differences in ATP synthesis capacity among populations of *T. californicus* and that these differences are likely involved in local thermal adaptation with latitude in this species, particularly over acute timescales of thermal exposure.

### Variation in Thermal Tolerance and Mitochondrial Functions Across Timescales

Several studies have demonstrated intraspecific differences in upper thermal tolerance with latitude in *T. californicus* that are consistent with local thermal adaptation from Mexico to Alaska (Willett, 2010; Kelly et al., 2012; Pereira et al., 2017). These studies employed experimental tests similar to our 1-h heat stress protocol, and our results generally corroborate previous findings for SD, AB, and SCN. Here, we also establish an alternative experimental method to examine variation in acute upper thermal tolerance in *T. californicus* through the use of knockdown temperatures, and we demonstrate that post-hoc tests with data from either of these thermal tolerance methods resolve similar inter-population patterns for variation in upper thermal limits (Figure 2). Our knockdown temperature assay is essentially similar to a critical thermal maximum assay (e.g., Beitinger et al., 2000) and provides several advantages such as reducing the number of animals required to measure upper thermal tolerance and allowing repeated tests of variation in upper thermal tolerance among individuals (e.g., Morgan et al., 2018). Therefore, this protocol is likely to facilitate future experiments investigating the genetic basis of variation in upper thermal limits in *T. californicus* and of local thermal adaptation more generally.

There is a growing body of evidence suggesting that collapse of ATP synthesis capacity is mechanistically involved in setting upper thermal limits during acute exposure to high temperature, particularly in fish hearts (Iftikar and Hickey, 2013; Christen et al., 2018; O’Brien et al., 2018). Cardiovascular failure due to arrhythmias is potentially a weak physiological link underlying upper thermal tolerance in fish (e.g., Farrell, 2009), and it is now thought that insufficient energy supply due to decreased mitochondrial ATP synthesis may underlie this heart failure (e.g., Iftikar and Hickey, 2013). Our results suggest that the loss of ATP synthesis capacity may also contribute to setting acute upper thermal limits in *T. californicus* (Figure 4), a species that does not rely on a heart to transport oxygen throughout the body. Rapid declines in CI&II-fueled ATP synthesis rate are observed at high temperatures in all three populations in our study, but these declines occur ~1°C lower in SCN than in AB or SD (~36 versus ~37°C; Figure 4A). In comparison, mean knockdown temperatures for the three populations are 36.5, 38.4, and 38.6°C for SCN, AB, and SD, respectively. Although the differences between the temperatures that result in high temperature knockdown or initial declines in ATP synthesis capacity vary somewhat among populations, declines in ATP synthesis rate occur at temperatures that are only slightly lower than knockdown temperatures in all cases. Furthermore, if our data are used to estimate CI&II-linked ATP synthesis rate for each population at knockdown temperatures, rates at upper thermal limits are similar regardless of population (0.89, 1.12, and 1.15 nmol min^−1^ mg mito. protein^−1^ for SD, AB, and SCN, respectively).

Here, we also demonstrate inter-population differences in the chronic effects of high temperature on survival and developmental rate in *T. californicus*. In the AB population, development at 25°C resulted in a decrease in survival over time when compared to development at 20°C, and this led to a significant difference in the effects of 25°C on developmental survival in AB compared to SD after 3 weeks (Figure 3B). Indeed, there was no evidence for a negative effect of 25°C on survival of development in SD copepods, and similarly, there was no significant effect of 25°C relative to 20°C in SCN. Somewhat contradicting these results for SD and SCN, Edmands and Deimler (2004) found negative effects of 25°C development relative to 15°C development in these populations; but consistent with our study, these authors found no difference between SD and SCN. Regardless, our data suggest that prolonged exposure to 25°C has greater negative effects on AB than on the other two populations in our study.

In all populations, development at 25°C, compared to 20°C, caused an increase in developmental rate (i.e., decrease in time to metamorphosis) that was consistent with temperature coefficients (Q_10_) of ~2 as would be expected (Hochachka and Somero, 2002); however, the increase in rate was smaller in AB than in either SD or SCN (Figure 3C). It is possible that this is simply a consequence of exponential effects of temperature on physiological rates and a lower 20°C rate in AB. Alternatively, there was evidence for reduced ATP synthesis capacity in AB compared to SD or SCN when substrates were provided to both CI and CII (Figure 4A) or to CI alone (Figure 4C), particularly at 25°C, which could result in trade-offs between development and other physiological costs when AB copepods undergo development at 25°C. Energetic compromises in life history traits in AB are also consistent with smaller egg clutches at 20°C than in the other populations and reduced ATP synthesis rates in AB copepods (at least compared to SCN copepods). The relationship between ATP synthesis rate, particularly under saturating substrate conditions, and energy demand at 20 and 25°C among populations is complex, as both ATP supply and demand are thermally sensitive and demand may vary among populations even at 20°C. However, our results may indicate that intraspecific variation in ATP synthesis capacity is related to thermal effects over prolonged timescales as well as the acute effects discussed above.

### Role of Variation in Mitochondrial Functions in Latitudinal Thermal Adaptation

Upper thermal limits in aquatic ectotherms decrease approximately linearly from the equator to the poles (Sunday et al., 2011) closely matching habitat temperatures (Sunday et al., 2012). Because *T. californicus* inhabits splash pools in the extreme upper intertidal, air temperatures are potentially better predictors of habitat temperature than sea surface temperatures for this species (e.g., Pereira et al., 2017), and differences in mean knockdown temperature among our populations (38.6, 38.4, and 36.5°C for SD, AB, and SCN, respectively; Figure 2) parallel the differences in mean annual air temperature between the populations (Pereira et al., 2017), although the differences in knockdown temperatures are smaller than those for estimated habitat temperatures. In part due to these similarities, variation in acute upper thermal tolerance is thought to be an important consequence of local thermal adaptation in this species (Willett, 2010; Kelly et al., 2012; Pereira et al., 2017). Thus, by extension, our data suggest that differences in the thermal sensitivity of ATP synthesis capacity (Figure 4) likely contribute to local adaptation across this latitudinal gradient. However, it is important to note that our study examined this trait in only three populations of *T. californicus* that are found across a relatively small proportion of the latitudinal range of the species (Mexico to Alaska), and therefore, the extent to which our results can be generalized to larger latitudinal ranges remains unclear and merits future experimental consideration.

The predominant signatures of selection that are typically detected for genes in the mitochondrial genome are those of purifying selection (Stewart et al., 2008; Palozzi et al., 2018), which is perhaps not surprising given the central roles many of these genes play in mitochondrial protein or RNA complexes. However, mitochondrial genes are often involved in adaptive responses (Ballard and Whitlock, 2004), and signatures of directional selection have been detected for some genes encoded in the mitochondrial genome in *T. californicus,* particularly CI genes (*nad3*, *nad5*, and *nad6*; Barreto et al., 2018). Furthermore, nuclear genes encoding products involved in mitochondrial functions have elevated nucleotide substitution rates compared to other nuclear genes in this species (Willett and Burton, 2004; Barreto et al., 2018). Given our results for latitudinal variation in the temperatures that result in the loss of ATP synthesis capacity among populations, it is possible that at least some of this genetic variation contributes to the differences in thermal sensitivity of ATP synthesis rate among populations.

In contrast to variation in upper thermal tolerance at acute timescales of exposure, differences in chronic effects of high temperature on survival or developmental rate were not clearly associated with latitudinal variation in temperature in the current study. Variation in life history traits (i.e., egg clutch size, developmental survival, and developmental rate) and changes in these traits due to elevated temperature were consistent with reduced survival and developmental rate in AB compared to SD and SCN (Figure 3). Although our data suggest that variation in ATP synthesis capacity may contribute to these differences (discussed above), these patterns are not obviously related to differences in habitat temperatures among SD, AB, and SCN. Thus, our data for these three populations do not support a role of mitochondria in latitudinal adaptation associated with prolonged thermal exposures (i.e., months or seasons) in *T. californicus*.

Variation in life history traits with latitude, and therefore environmental temperature, is common in ectotherms (e.g., Chung et al., 2018b), and *T. californicus* is not an exception to this trend (Hong and Shurin, 2015). Latitudinal changes in these traits in *T. californicus* are smooth and gradual, such that relatively little variation is expected along the Californian coast in general (Hong and Shurin, 2015), which is consistent with our results in SD and SCN, which span less than half of California’s latitudinal range. Therefore, it is possible that comparisons of more geographically distant populations than those used in the current study would reveal variation in chronic temperature effects on ATP synthesis capacity that parallel differences in habitat temperatures.

Taken together, the data presented in the current study suggest a role for mitochondrial functions, particularly ATP synthesis capacity, in determining the limits of tolerance of both short- and long-term exposures to elevated temperatures. Variation in acute upper thermal tolerance in *T. californicus* is consistent with local thermal adaptation (Willett, 2010; Kelly et al., 2012; Pereira et al., 2017; the current study), and therefore, our results suggest that divergence in ATP synthesis capacity is a component of adaptation across latitudinal thermal gradients as well. Given that the acute effects of temperature in our study more closely paralleled habitat temperatures than chronic effects of temperature, our data also suggest that extreme temperature events impose important selection pressures that likely drive local thermal adaptation, as has been suggested previously (Somero, 2010; Siegle et al., 2018). Determining both the extent to which thermal sensitivity of ATP synthesis capacity influences the effects of these rare events on organisms and the mitochondrial mechanisms that underlie this sensitivity will be critical steps in accurately predicting the impacts of future environmental change on ectotherms.

## Author Contributions

All authors listed have made a substantial, direct and intellectual contribution to the work, and approved it for publication.

### Conflict of Interest Statement

The authors declare that the research was conducted in the absence of any commercial or financial relationships that could be construed as a potential conflict of interest.
