# Supplementary material for: Variation in Thermal Tolerance and Its Relationship to Mitochondrial Function Across Populations of Tigriopus californicus
Source: Front Physiol. 2019 Mar 15;10:213. doi: 10.3389/fphys.2019.00213 (PMC6429002; doi:10.3389/fphys.2019.00213)
Supplement: Supplementary file 1 [file Table_1.DOCX]

**Table S1**. Proportion of nauplii that survived or metamorphosed (mean ± SEM), and proportion of clutches with adult males throughout development at 20 or 25 °C.

|  | **Time (d)** | **San Diego (SD)** | | **Abalone Cove (AB)** | | **Santa Cruz (SCN)** | |
| --- | --- | --- | --- | --- | --- | --- | --- |
|  |  | **20 °C** | **25 °C** | **20 °C** | **25 °C** | **20 °C** | **25 °C** |
| **Survival proportion** | 7 | 0.97 ± 0.01 | 1.00 ± 0.00 | 0.96 ± 0.03 | 0.95 ± 0.02 | 0.97 ± 0.01 | 0.90 ± 0.03 |
|  | 14 | 0.96 ± 0.01 | 0.96 ± 0.02 | 0.96 ± 0.03 | 0.88 ± 0.04 | 0.95 ± 0.02 | 0.84 ± 0.03 |
|  | 21 | 0.92 ± 0.03 | 0.94 ± 0.03 | 0.89 ± 0.04 | 0.69 ± 0.07 | 0.92 ± 0.02 | 0.82 ± 0.03 |
| **Metamorphosis proportion** | 7 | 0.86 ± 0.06 | 0.96 ± 0.02 | 0.90 ± 0.05 | 0.99 ± 0.01 | 0.95 ± 0.02 | 0.99 ± 0.01 |
|  | 14 | 1.00 ± 0.00 | 0.99 ± 0.01 | 0.98 ± 0.02 | 1.00 ± 0.00 | 1.00 ± 0.00 | 1.00 ± 0.00 |
|  | 21 | 1.00 ± 0.00 | 1.00 ± 0.00 | 0.99 ± 0.01 | 1.00 ± 0.00 | 1.00 ± 0.00 | 1.00 ± 0.00 |
| **Proportion of clutches with adult males** | 7 | 0.00 | 0.00 | 0.00 | 0.00 | 0.00 | 0.00 |
|  | 14 | 0.00 | 0.58 | 0.00 | 0.38 | 0.00 | 0.25 |
|  | 21 | 0.67 | 1.00 | 0.38 | 0.83 | 0.88 | 0.96 |

**Table S2**. ANOVA table for survivorship following 1-h acute heat stress (data presented in Figure 2A).

| **Factor** | **df** | **Resid. df** | **Resid. dev.** | **χ^2^** | ***p*** |
| --- | --- | --- | --- | --- | --- |
| Population | 2 | 69 | 540.87 | 60.38 | 7.7 x 10^-14^ |
| Temperature | 3 | 66 | 217.73 | 322.13 | <2.2 x 10^-16^ |
| Interaction | 6 | 60 | 189.61 | 29.12 | 5.8 x 10^-5^ |
| NULL |  | 71 | 601.25 |  |  |

**Table S3**. ANOVA table for knockdown temperatures (data presented in Figure 2B).

| **Factor** | **df** | **SS** | **F** | ***p*** |
| --- | --- | --- | --- | --- |
| (Intercept) | 1 | 23593.0 | 44062.38 | <2.2 x 10^-16^ |
| Population | 2 | 44.5 | 41.52 | 6.1 x 10^-11^ |
| Residuals | 45 | 24.1 |  |  |

**Table S4**. ANOVA table for number of eggs per clutch (data presented in Figure 3A).

| **Factor** | **df** | **SS** | **F** | ***p*** |
| --- | --- | --- | --- | --- |
| (Intercept) | 1 | 37447 | 1283.09 | <2.2 x 10^-16^ |
| Population | 2 | 2472 | 42.34 | 1.0 x 10^-12^ |
| Residuals | 69 | 2014 |  |  |

**Table S5**. ANOVA table for differences in developmental survival at 20 and 25 °C (data presented in Figure 3B).

| **Factor** | **df** | **χ^2^** | ***p*** |
| --- | --- | --- | --- |
| (Intercept) | 1 | 5.71 | 1.7 x 10^-2^ |
| Population | 2 | 5.94 | 5.1 x 10^-2^ |
| Time | 2 | 29.18 | 4.6 x 10^-7^ |
| Interaction | 4 | 16.50 | 2.4 x 10^-3^ |

**Table S6**. Kruskal-Wallis ANOVA table for variation in time to metamorphosis among treatments (population x temperature; data presented in Figure 3C).

| **Factor** | **df** | **χ^2^** | ***p*** |
| --- | --- | --- | --- |
| Treatment | 5 | 681.73 | <2.2 x 10^-16^ |

**Table S7**. ANOVA table for ATP synthesis rate fueled by CI&II substrates (data presented in Figure 4A).

| **Factor** | **df (numerator)** | **df (denominator)** | **F** | ***p*** |
| --- | --- | --- | --- | --- |
| (Intercept) | 1 | 120 | 227.33 | <1 x 10^-4^ |
| Population | 2 | 15 | 0.30 | 0.75 |
| Temperature | 8 | 120 | 104.99 | <1 x 10^-4^ |
| Interaction | 16 | 120 | 7.42 | <1 x 10^-4^ |

**Table S8**. ANOVA table for ATP synthesis rate fueled by CI substrates (data presented in Figure 4B).

| **Factor** | **df (numerator)** | **df (denominator)** | **F** | ***p*** |
| --- | --- | --- | --- | --- |
| (Intercept) | 1 | 120 | 60.63 | <1 x 10^-4^ |
| Population | 2 | 15 | 2.67 | 0.10 |
| Temperature | 8 | 120 | 46.69 | <1 x 10^-4^ |
| Interaction | 16 | 120 | 4.48 | <1 x 10^-4^ |

**Table S9**. ANOVA table for ATP synthesis rate fueled by CII substrates (data presented in Figure 4C).

| **Factor** | **df (numerator)** | **df (denominator)** | **F** | ***p*** |
| --- | --- | --- | --- | --- |
| (Intercept) | 1 | 120 | 134.99 | <1 x 10^-4^ |
| Population | 2 | 15 | 0.60 | 0.56 |
| Temperature | 8 | 120 | 48.78 | <1 x 10^-4^ |
| Interaction | 16 | 120 | 2.32 | 5.1 x 10^-3^ |

**Table S10**. ANOVA table for state III respiration rates (data presented in Figure 5A).

| **Factor** | **df** | **SS** | **F** | ***p*** |
| --- | --- | --- | --- | --- |
| (Intercept) | 1 | 341.24 | 8806.39 | <2 x 10^-16^ |
| Population | 2 | 0.06 | 0.76 | 0.47 |
| Temperature | 1 | 0.01 | 0.32 | 0.57 |
| Interaction | 2 | 0.13 | 1.64 | 0.21 |
| Residuals | 48 | 1.86 |  |  |

**Table S11**. ANOVA table for state IV_ol_ respiration rates (data presented in Figure 5B).

| **Factor** | **df** | **SS** | **F** | ***p*** |
| --- | --- | --- | --- | --- |
| (Intercept) | 1 | 248.05 | 2929.24 | <2 x 10^-16^ |
| Population | 2 | 0.15 | 0.87 | 0.43 |
| Temperature | 1 | 0.40 | 4.74 | 3.4 x 10^-2^ |
| Interaction | 2 | 0.09 | 0.55 | 0.58 |
| Residuals | 48 | 4.07 |  |  |

**Table S12**. ANOVA table for respiratory control ratios (data presented in Figure 5C).

| **Factor** | **df** | **SS** | **F** | ***p*** |
| --- | --- | --- | --- | --- |
| (Intercept) | 1 | 410.77 | 156.74 | <2 x 10^-16^ |
| Population | 2 | 9.03 | 1.72 | 0.19 |
| Temperature | 1 | 12.24 | 4.67 | 3.6 x 10^-2^ |
| Interaction | 2 | 7.53 | 1.44 | 0.25 |
| Residuals | 48 | 125.79 |  |  |
